# Reactivity of Single-Atom Alloy Nanoparticles: Modeling the Dehydrogenation of Propane

**DOI:** 10.1021/jacs.3c04030

**Published:** 2023-06-30

**Authors:** Rhys J. Bunting, Felix Wodaczek, Tina Torabi, Bingqing Cheng

**Affiliations:** † Institute of Science and Technology Austria, Am Campus 1, 3400 Klosterneuburg, Austria

## Abstract

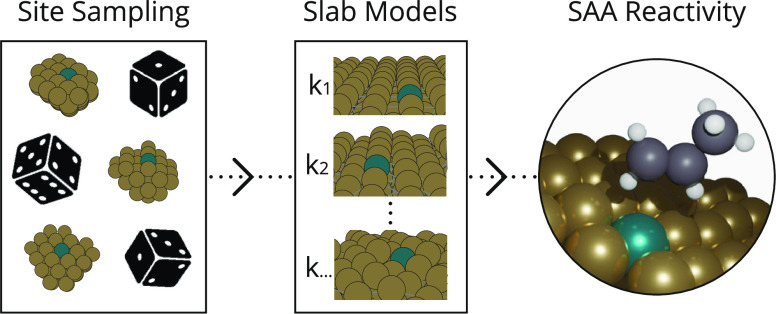

Physical catalysts often have multiple sites where reactions can take place. One prominent example is single-atom alloys, where the reactive dopant atoms can preferentially locate in the bulk or at different sites on the surface of the nanoparticle. However, ab initio modeling of catalysts usually only considers one site of the catalyst, neglecting the effects of multiple sites. Here, nanoparticles of copper doped with single-atom rhodium or palladium are modeled for the dehydrogenation of propane. Single-atom alloy nanoparticles are simulated at 400–600 K, using machine learning potentials trained on density functional theory calculations, and then the occupation of different single-atom active sites is identified using a similarity kernel. Further, the turnover frequency for all possible sites is calculated for propane dehydrogenation to propene through microkinetic modeling using density functional theory calculations. The total turnover frequencies of the whole nanoparticle are then described from both the population and the individual turnover frequency of each site. Under operating conditions, rhodium as a dopant is found to almost exclusively occupy (111) surface sites while palladium as a dopant occupies a greater variety of facets. Undercoordinated dopant surface sites are found to tend to be more reactive for propane dehydrogenation compared to the (111) surface. It is found that considering the dynamics of the single-atom alloy nanoparticle has a profound effect on the calculated catalytic activity of single-atom alloys by several orders of magnitude.

## Introduction

Catalysis contributes to a third of the global economy and is crucial across several fields of science.^1^ Unsurprisingly, much focus has been placed on the theoretical modeling of catalytic systems.^2^ The goal of these models can vary from understanding a catalytic system to developing a new catalyst adept for a reaction process. Density functional theory (DFT) is the most extensively used method to study surface science from an ab initio perspective, offering a good balance between computational cost and chemical accuracy.^3^ Slab models are the standard within heterogeneous catalysis, where a certain cleave/surface of the bulk phase is considered. Often, the most stable facet is considered for a key elementary step of a process to describe the reactivity of a catalyst. This does not give a full description of the catalytic system outside of single-crystal catalysts.^4^

One of the most widely used classes of catalysts are metal-based catalysts. Most metal-based catalysts are dispersed nanoparticles of varying sizes.^5^ Besides the most stable facet, these metal nanoparticles can have a variety of available facets and sites for reactions to take place. Each distinct site has its own reactivity and contributes to the overall reactivity of a nanoparticle.^6^ Desirably, a theoretical model would offer a complete understanding of the catalyst as a whole, where all sites on the surface are modeled. However, theoretical models that employ DFT are limited by the system size, with typically cubic scaling of the computational cost with respect to the number of electrons.^3^ In real terms, metal slab models in catalysis are limited to ∼100 atoms; metal nanoparticles can range in size from ∼200 to 100,000+ atoms. New methods are required to capture the reactivity of an experimentally representative nanoparticle catalyst.

Machine learning (ML) potentials have been demonstrated as effective tools to describe very large atomic systems with equivalent accuracy to ab initio methods.^7^ ML potentials can therefore overcome the technical limitations of DFT calculations, enabling experimentally sized nanoparticles to be considered over extended timescales. ML potentials have effectively been used to study hydrogen coupling,^8^ CO oxidation,^9^ CO_2_ reduction,^10^ and ethanol reforming.^11^

Single-atom alloys (SAAs) are a unique class of metal–alloy-based catalysts, where the dopant constitutes a small amount (∼1%) of the alloy.^12,13^ The dopant is dilute and dispersed enough throughout the host metal lattice so as to not interact with other dopant atoms. For traditional alloys, dopants usually influence the properties of the host metal, changing the reactivity of the surface. This is counter to SAAs, where the chemical reaction takes place effectively exclusively at the dopant site; SAAs have localized active centers on the surface of the catalyst.^14^ Localized reactions offer unique selectivities specific to this special class of metal catalysts, such as the punctured molecular cork effect.^15^ The key advantage of SAAs is their ability to break linear scaling relationships, increasing both reactivity and selectivity.^16,17^ The potential of this effect has been shown for selective methane activation,^18^ dehydrogenation of ethanol,^19^ selective acetylene hydrogenation,^20^ and selective propane dehydrogenation.^21^ Propane dehydrogenation to propene is a challenging process due to selectivity requirements.^22−24^ Excess dehydrogenation of the propene product is undesirable from not only a yield perspective but also from a catalyst deactivation perspective, as it can lead to carbon coking of the surface which will deactivate the surface. Rh–Cu and Pt–Cu SAAs have shown promise of enhanced dehydrogenation selectivity which minimizes coke formation.^21,25,26^ In addition to their selectivity, considerable design space is available with the mixture of different host metals and dopant metals.^13^

Dopant metals can diffuse to a variety of sites on the surface or into the bulk of the nanoparticle, with different SAA dopant–host combinations having varying propensities for the dopant to be more stable in the bulk or surface sites.^21^ This contributes to the difficulty of modeling the reactivity of a single-atom alloy, as not only the chemical reactivity of certain sites needs to be considered but also the dopant occupation of sites on the surface. The dynamics of the catalyst are crucial in the overall reactivity of the catalyst.

Herein, the reactivity of experimentally representative SAA nanoparticles is considered using ML methods and DFT microkinetic modeling. In this work, we train an equivariant neural network potential for Rh–Cu and Pd–Cu SAAs using DFT training data; classify sites on the surface of a nanoparticle; discern the probability of finding the dopant atom in each type of site by performing molecular dynamics; model the reactivity of each possible site through microkinetic modeling using DFT; and, ultimately, determine the total reactivity of both Rh–Cu and Pd–Cu SAA nanoparticle catalysts for the dehydrogenation of propane as a function of temperature.

## Methods

### Density Functional Theory Calculations

Density functional theory calculations were performed with the Perdew–Burke–Ernzerhof (PBE)^27^ functional within the generalized gradient approximation (GGA) in the Vienna Ab initio Simulation Package (VASP).^28−30^ The D3 dispersion correction with Becke–Johnson damping was also applied to consider van der Waals forces.^31^

The projector-augmented wave (PAW) method was used to represent the core–valence electron interaction.^32,33^ Structures were optimized until the forces on all atoms were below 0.05 eV Å^–1^. Transition states were searched with the constrained minimization technique and verified with a vibrational frequency calculation, where no imaginary frequencies were identified as an initial state and one imaginary frequency as a transition state.^34,35^

For the DFT calculations of the metal surfaces, the cutoff energy of the plane-wave basis set was 450 eV. The Brillouin zone was sampled using the Monkhorst–Pack scheme with a 5 × 5 × 1 *k*-point mesh for all surfaces, a 5 × 5 × 5 grid for bulk metal, and to the gamma point for molecules.^36^ A vacuum layer of at least 12 Å was also used for all surfaces. Gas-phase molecules were calculated in a 25 × 25 × 25 Å^3^ box to prevent periodic interactions.

For the dataset DFT calculations, the following models were used: A 3 × 3 × 3 supercell was used for bulk metal; a *p*(4 × 4) slab was used for the (100) surface; a *p*(3 × 4) slab for the (110) surface; an orthorhombic *p*(4 × 3) slab for the (111) surface; and a *p*(2 × 4) slab for the stepped (211) surface. For the surfaces, all slabs were 5 layers thick. A larger supercell was used for the ML potential dataset, so structures with a ∼1% dopant loading could be considered.

For the microkinetic modeling DFT calculations, the following models were used: all slabs were 4 layers thick with the bottom 2 layers fixed; a *p*(3 × 3) slab was used for the (100) surface; a *p*(2 × 3) slab for the (110) surface; an orthorhombic *p*(3 × 2) slab for the (111) surface; and a *p*(1 × 3) slab for the (211) surface. Single-atom alloys were modeled as one surface copper atom replaced in the unit cell with the defined dopant atom. All unique sites were modeled on these surfaces through adding adatoms or making surface vacancies.

### Molecular Dynamics Calculations

All molecular dynamics calculations were performed within the LAMMPS package.^37^ Transmutation Monte Carlo swap simulations were performed for 100,000 swaps at each temperature and equilibrated for 10,000 swaps. Molecular dynamics calculations were performed within the NVT ensemble with the Nosé–Hoover thermostat.^38^ A step size of 1 fs was used. For the molecular dynamics with transmutation Monte Carlo swap,^39^ simulations were run for 1,000,000 time steps, with atom swapping being performed every 100 steps for 15 atoms, and equilibrated for 10,000 steps. Each time step was structurally optimized to a minimum with a force criterion of 0.05 eV Å^–1^ before surface site classification was performed. The more occupied a site is, the more favorable its formation is.

An initial dataset was generated with molecular dynamics calculations within the NVT ensemble using an embedded atom model^40^ for bulk copper and the various slabs. Snapshots were taken of these simulations, and 2–3 copper atoms were swapped with respective dopant atoms dependent on the SAA considered. Single-point-energy DFT calculations were performed, and an ML potential was trained for each SAA.

The final dataset was generated through molecular dynamics with transmutation Monte Carlo swap simulations with the initial ML potentials. Bulk metal, 5 layer thick relaxed slab models ((100)/(110)/(111)/(211) surfaces) were considered. A range of 1–3 atoms in the various structures were replaced with dopants in the dataset. Calculations were performed at 200, 500, and 1200 K.

For bulk metal and surfaces, 1800 structures were generated for each category. Within this, 600 are at each defined temperature, and at each temperature, 200 are with 1 dopant, 200 with 2 dopants, and 200 with 3 dopants. In total, there are 9000 structures in each dataset for each SAA.

### Machine Learning Potential Training

The ML potential was trained using the NequIP package.^41^ The architecture of the network uses 3 interaction blocks. 32 features are considered with a maximum rotation order of *l* = 1. The radial network uses 8 basis functions with a polynomial cutoff *p*-exponent of 6. The radial network architecture has 2 layers with 64 neurons. A cutoff radius of 4 Å was used. 8640 randomly selected structures were used for the training set (90%), and the other 960 structures (10%) are used for the validation set.

### Smooth Overlap of Atomic Positions Descriptor Generation and Comparison

All smooth overlap of atomic positions (SOAP) descriptors used were generated with the DScribe package.^42^ A cutoff distance of 5.2 Å was used with 4 spherical Gaussian-type orbitals as radial basis functions and spherical harmonics with *l*_max_ = 3 for mapping the Gaussian-type density functions with σ = 1 around the atom positions. Upon comparing SOAP descriptors, the radial basis function pairwise comparison kernel implemented in the DScribe package used σ = 1.

### Free Energy Pathways and Microkinetic Modeling

For the free energy calculations and microkinetic modeling, zero-point energy corrections were applied to all surface species and gas molecules. The entropic contributions of gas molecules were considered with the ideal gas approximation.^43^ Adsorbed surface species were modeled as having restricted translational and rotational entropic contributions on the surface. Only vibrational entropic contributions were considered within the harmonic approximation.^43^ Free energy barriers for the forward and reverse reactions of each elementary step were calculated for the temperatures of 400, 500, and 600 K under standard pressure. Energy barriers were set to 0.05 eV if a lower barrier than this value is calculated to remove numerical issues. Barriers of adsorption/desorption steps were also set to be 0.05 eV.

For the microkinetic modeling, the considered steps are:

1. C_3_H_8(g)_ + * → C_3_H_8_*
2. C_3_H_8_* → C*H_2_CH_2_CH_3_ + H*
3. C_3_H_8_* → CH_3_C*HCH_3_ + H*
4. C*H_2_CH_2_CH_3_ + H* → C_3_H_6_* + 2H*
5. CH_3_C*HCH_3_ + H* → C_3_H_6_* + 2H*
6. C_3_H_6_* + 2H* → C_3_H_6(g)_ + 2H*
7. 2H* → H_2_*
8. H_2_* → H_2(g)_ + *

The diffusion of species between sites was assumed to be negligible. Standard pressures of 0.014 bar of propane and 0.007 bar of hydrogen were selected for the conditions. The system was considered to be under a steady state for the calculated rates. The steady state was found by solving the series of differential equations correlating the rates of each elementary step. A variable-coefficient ordinary differential equation solver, implemented in the SciPy package, was used to solve the concentration of surface species.^44^

## Results and Discussion

### ML Potentials for Rh–Cu and Pd–Cu SAAs

The training datasets of the ML potentials are made from snapshots of classical molecular dynamics with transmutation Monte Carlo swap simulations of bulk doped copper and slab models of the doped Cu(100), Cu(110), Cu(111), and Cu(211) surfaces. For this, an initially generated ML potential is used. The dopant is either Rh or Pd, dependent on the SAA that is modeled. Rh is selected as a dopant due to a Rh–Cu SAA catalyst being used for propane dehydrogenation.^21^ Pd is selected as a dopant as Pd–Cu SAA catalysts have successfully been used in a variety of reactions.^45−49^ Single point energies are calculated for the dataset with DFT, using the PBE functional with the D3(BJ) dispersion correction.^50,51^ This dataset is used for the training of an equivariant neural network interatomic potential using the NequIP package.^41^

The final trained ML potential for the Rh–Cu SAA has a root-mean-squared training error of 0.53 meV per atom for energy and 14.5 meV Å^–1^ for all force components. For the Pd–Cu SAA ML potential, the error is 0.46 meV per atom for energy and 12.7 meV Å^–1^ for all force components. To validate the ML potentials, static DFT calculations are performed (using the same exchange–correlation functional used to calculate the dataset) and compared to static calculations using the ML potentials. The bulk lattice constant of copper is equivalent for both DFT and the ML potentials (3.56 Å). Additionally, the total energy change of the dopant atom moving from the bulk of the nanoparticle to the (100), (110), or (111) surface with the ML potential matches closely within 0.05 eV with respect to slab calculations using DFT (SI.1). This discrepancy can be explained by the change of surface stress going from a slab to a nanoparticle model.

### Site Classification of SAA Nanoparticles

**Figure 1:**
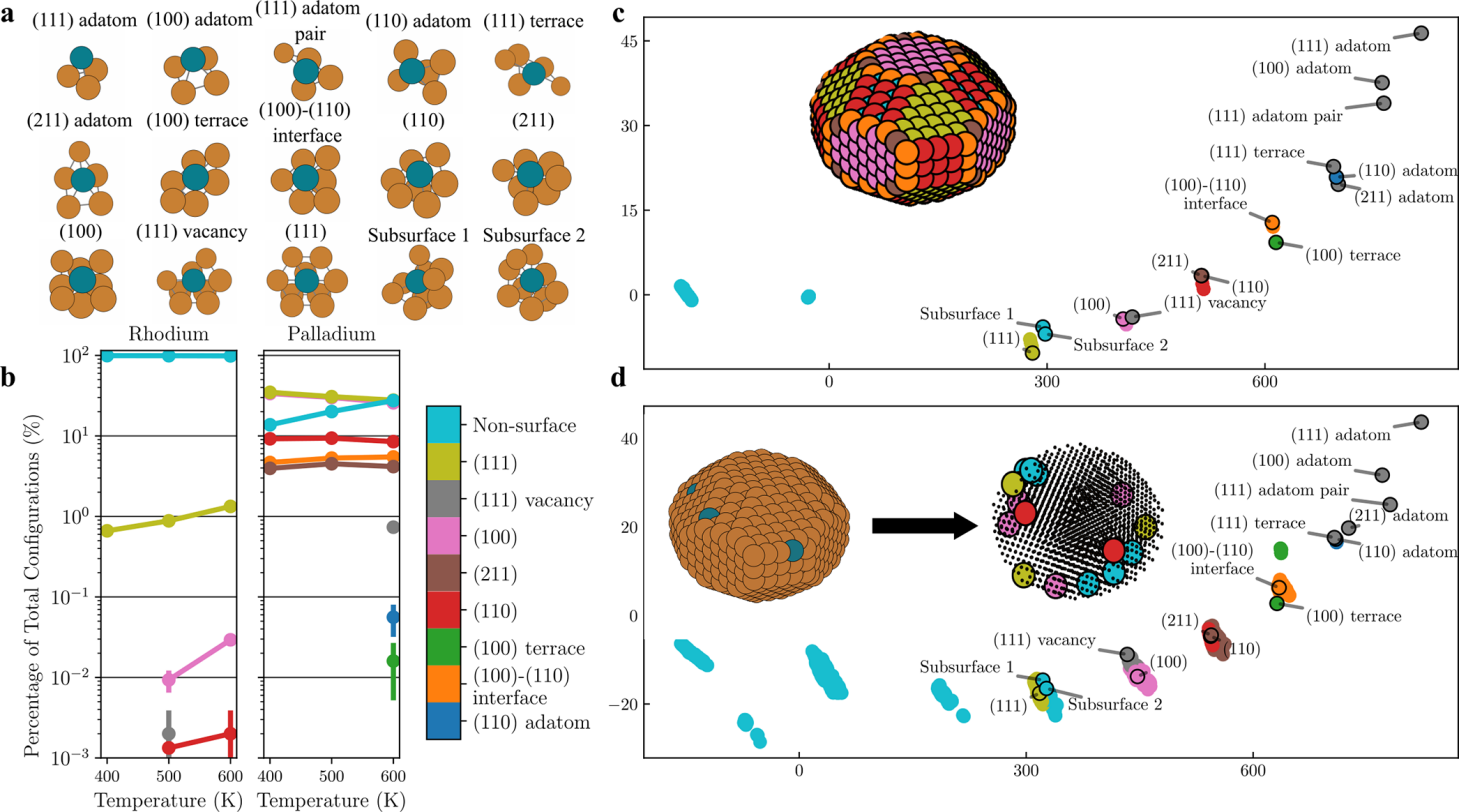
Classification and dopant occupation of different sites on SAA nanoparticles. (a) The dictionary of possible sites when the first nearest neighbor in the FCC lattice structure is considered. Structures of how these sites can appear on the nanoparticle surface are shown in SI.2.2. (b) The percent likelihood for a dopant atom to occupy a specific surface in the nanoparticle calculated by molecular dynamics calculations with transmutation Monte Carlo swap at 400–600 K, using the generated ML potentials. All surface sites are categorized, denoted by their color, shown in the color bar. All non-surface sites are merged into one category (non-surface). (c) Principle component analysis (PCA) map of SOAP descriptors from dictionary entries for classification and from an optimized sample copper nanoparticle. The sample nanoparticle is shown in the inset plot with the sites marked according to their classification. (d) PCA map of SOAP descriptors for a constructed Pd–Cu site dictionary and for a molecular dynamics trajectory with transmutation Monte Carlo swap of Pd–Cu at 600 K. The inset shows a single snapshot of the trajectory. Before classification (left), the copper sites are marked reddish-brown and the palladium sites are marked steel blue. After classification (right), only the palladium atoms are colored according to their site classification.

A copper nanoparticle is made using a Wulff construction based on the surface energies of the experimentally most stable surfaces^52^ ((111), (100), (110)), as seen in Figure 1c,d. The nanoparticle size is set to 3 nm in diameter, as found in experimental work where a Rh–Cu SAA catalyst is used for the dehydrogenation of propane.^21^ Many distinct single-atom sites are present on the surface. If only the first nearest neighbors of the single-atom sites are considered, there are 5 surface sites. These correspond to the (100) surface site, (110) surface site, (111) surface site, (211) surface site, and (100)–(110) interface site. There are also two non-surface sites, equating to the (110) subsurface and bulk lattice sites. However, this only describes the sites available on the ordered most stable nanoparticle. When all possible local environments are considered, derived from the cleaving of the face-centered cubic (FCC) crystal structure, 13 possible surface sites are found, as listed and shown in Figure 1a.

Classification of all possible sites is required to designate which site the SAA dopant atom occupies during a simulation. An initial classification is done by counting the number of neighbors the single-atom site has. From 10 to 12 neighbors, the single-atom site is either part of the subsurface (10 and 11 neighbors) or bulk lattice (12 neighbors). These are classified as non-surface sites. For 9 neighbors, the site is either the (111) surface or two possible subsurface (non-surface) sites. Between 3 and 8 neighbors (where 3 neighbors is the minimum number of neighbors possible for a local minima structure on the surface), a variety of surface sites are available and must be classified.

A dictionary of the possible structures for 3–9 neighbor single-atom sites is constructed manually, as shown in Figure 1a, where the center of the site (the single atom) is designated by a steel blue color. The local environments of the central single atoms in the dictionary are represented by the SOAP^53^ descriptor as implemented in the DScribe package.^42^ SOAP descriptors are capable of describing the similarity of different atomic environments.^53^ Local structures (to the first neighbor) of all atoms are made from neighbor lists as implemented in the atomic simulation environment (ASE) package.^43^ SOAP descriptors are then made for the central atom of all atomic local structures within the nanoparticle that have 3–9 neighbors. SOAP descriptors of the local atomic environments are compared to the SOAP descriptors of the dictionary. The sites are classified using a radial basis function pairwise comparison kernel between two sets of SOAP descriptors ***x***_1_ and ***x***_2_ defined as
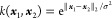
1where ∥·∥_2_ is the Euclidean distance and σ is the width of the Gaussian distribution. Equation 1, as implemented in DScribe,^42^ is used to attain similarity kernel values between a local environment and all of the dictionary entries of the nanoparticle sites. The comparison is made for all dictionary entries with the same number of nearest neighbors as the selected site. The dictionary entry with the highest similarity kernel value is chosen as the classification for that site.

A principle component analysis (PCA) map^54^ is built from all SOAP descriptors originating from the local environments of every single atom in the ML potential optimized Wulff-constructed copper nanoparticle (Figure 1c) and the molecular dynamics with transmutation Monte Carlo swap trajectories of dopant atoms for the Pd-doped nanoparticle (Figure 1d). The first two principal axes show clear clustering of the existing sites around the dictionary entries. There is some spread of the SOAP descriptors within classes. This is due to the local structures of atoms of the same nearest-neighbor site classification varying slightly. For example, the (100) facet has 21 (100) surface sites if only the first nearest neighbors are considered. Some are closer to corners and edges, giving slightly different geometries and therefore slightly different local environments.

The clustering of sites on the PCA map in Figure 1c proves that all sites that occur on an ML potential optimized Wulff-constructed copper nanoparticle, as shown in Figure 1c (top left), are represented in the dictionary. This clustering allows classification of the sites, with the classification being able to be checked through the visualization on the nanoparticle. Figure 1d serves as a proof of concept of the classification, visualizing all Pd sites in 10,000 snapshots of a molecular dynamics with transmutation Monte Carlo swap simulation of a Pd-doped copper nanoparticle at 600 K, as well as an example frame of the trajectory with Pd sites marked according to their classification. The color bar in Figure 1 lists only the sites that are found during the trajectory of the nanoparticle, making up 10 of the 15 sites in the dictionary (where subsurface 1 and subsurface 2 are combined with the non-surface sites). There are more non-surface sites present on the map due to the formation of new 10/11 neighbor defect sites, seen in light blue left of the (111) site dictionary entry. Further, there is more smearing in Figure 1d as the nanoparticle optimizes again at every timestep of the trajectory to a slightly different structure within the force convergence criteria compared to the nanoparticle shown in Figure 1c. Despite the additional smearing in the molecular dynamics with transmutation Monte Carlo swap trajectory, the Pd sites stay within the range of the dictionary entries on the PCA map and are classified correctly by using the pairwise comparison of SOAP descriptors. In Figure 1c,d, there is clear clustering of the sites found on the nanoparticle around the dictionary entries.

### Modeling SAA Nanoparticles with a Variety of Sites

With all sites on a nanoparticle being classifiable, the dynamics of the sites on the SAA nanoparticles can be quantified. Initial structures for SAA nanoparticles for Rh–Cu and Pd–Cu are made by randomly replacing Cu atoms within the Wulff-constructed nanoparticle with the typical experimental percentage of dopant atoms (∼1%, 15/1577 atoms) for SAAs. While the dopant loading is kept constant to allow comparison with experimental work, changing the dopant loading could influence the surface sites that form, including aggregation of dopant atoms, as seen in other work.^55^ To consider the effects of finite temperature, as in the local structural relaxation, thermal vibrations, and expansion of the nanoparticle with respect to temperature, along with the formation of adatoms and other defects, molecular dynamics with transmutation Monte Carlo swap calculations are performed. Atom swapping of dopant atoms with copper is performed to sample the slow diffusion process of dopant atoms moving throughout the lattice. Snapshots from the simulations are first optimized to a local minimum and then classified to minimize misclassification. The results of the classification are shown in Figure 1b for both rhodium and palladium at 400, 500, and 600 K. The results are compared with static transmutation Monte Carlo swap calculations to sanity check the classification (SI.2).

Both systems modeled are described by the simulations as SAAs, matching experimental work.^21,45−49^Figure 1b shows that almost all of the dopant atoms occupy bulk sites in the Rh–Cu nanoparticle, while most take surface sites for Pd–Cu. This key difference between Rh and Pd as dopants is due to Rh having a propensity for the bulk (0.06 eV ML potential nanoparticle) with respect to the (111) surface site, while Pd has a propensity for the surface (−0.18 eV ML potential nanoparticle). Upon increasing the temperature, two different effects can be seen for the two SAAs in (Figure 1b). For Rh as a dopant, more bulk dopant sites move to surface sites; for Pd, more surface sites move to the bulk, visible in Figure 1b. This can again be explained by the preference for the dopant to occupy the bulk or the surface. For rhodium, the diffusion of the dopant atom from the bulk to the surface is endothermic. With higher temperatures, more thermal energy is available to drive the equilibrium toward surface sites. For palladium, the diffusion of the dopant atom is exothermic for the main (111), (100), and (110) facets. When temperature increases, the equilibrium shifts toward less dopant surface sites forming for these facets. However, for the very undercoordinated surface sites for palladium, such as the (100)–(110) interface, diffusion is not favored. When temperature increases, the equilibrium shifts toward more of these sites forming. Pd–Cu has ∼130× more surface sites than Rh–Cu at 400 K. This aspect would make Pd–Cu a more reactive catalyst than Rh–Cu if the reactivity of the Pd/Rh dopant sites were all equivalent in their local reactivity.

Across all temperatures for simulations, for both Rh–Cu and Pd–Cu, the most populated surface site for the dopant is the (111) terminal site (Figure 1b). The (111) terminal site being the most populated is expected, as for both Rh and Pd, the most stable surface site for the dopant is the (111) terminal site at the ML potential and DFT level (SI.1). Some sites are considerably less stable than the dominant bulk or surface sites, such as the (110) site for Rh and the (100)–(110) interface site for Pd. For both dopants, when temperature increases, more unstable sites become available. The stability of the surface sites can generally be ordered by their number of neighbors, observed by the population of different sites with increasing temperature. At higher temperatures, less stable sites become populated, such as the (110) terminal site (7 neighbors), the (211) step site (7 neighbors), and the (100)–(110) interface step site (6 neighbors).

The reactivity of a catalyst can, in an overly simplistic way, be increased if the number of active sites is increased. More sites are present upon increasing the temperature for Rh–Cu for all site classifications; temperature makes Rh–Cu SAA nanoparticles more reactive (Figure 1b, left). For Pd–Cu, a higher temperature reduces the total number of available sites, reducing the number of (111) terminal dopant surface sites with an increase in the amount of bulk dopant atoms (Figure 1b, right). However, the number of undercoordinated sites, such as the (110) terminal site or the (110)–(100) interface sites, increases. Crucially, undercoordinated metal surface sites have been found to be more active for C–H activation in other work.^56^ While the total number of active sites decreases with temperature, more active sites become populated.^57^ With temperature being able to promote the formation of surface sites, it could be possible to synthesize or heat single-atom alloys to higher temperatures and then rapidly cool them. The kinetics of diffusion could limit the return of the nanoparticle to surface site equilibrium, with less surface sites, at that temperature. This may allow the formation of more reactive SAA catalysts for reactions at low temperatures.

### Reactivity of Individual Surface Sites

The rate constant for the dehydrogenation of propane can be calculated for each site, for both Rh–Cu and Pd–Cu, through microkinetic modeling. The turnover frequency (TOF) of an SAA catalyst with *N* sites at finite temperature *T* is defined as
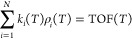
2where *k*_*i*_(*T*) is the overall rate constant for a surface site and ρ_*i*_(*T*) is the corresponding probability that the dopant atom occupies that site in the nanoparticle. The probability of each site being occupied is found from the performed calculations in Figure 1b. Microkinetic modeling is performed at the DFT level with the exchange–correlation functional that was used to train the ML potential (PBE-D3(BJ)) to find the overall rate constant of each site.

**Figure 2:**
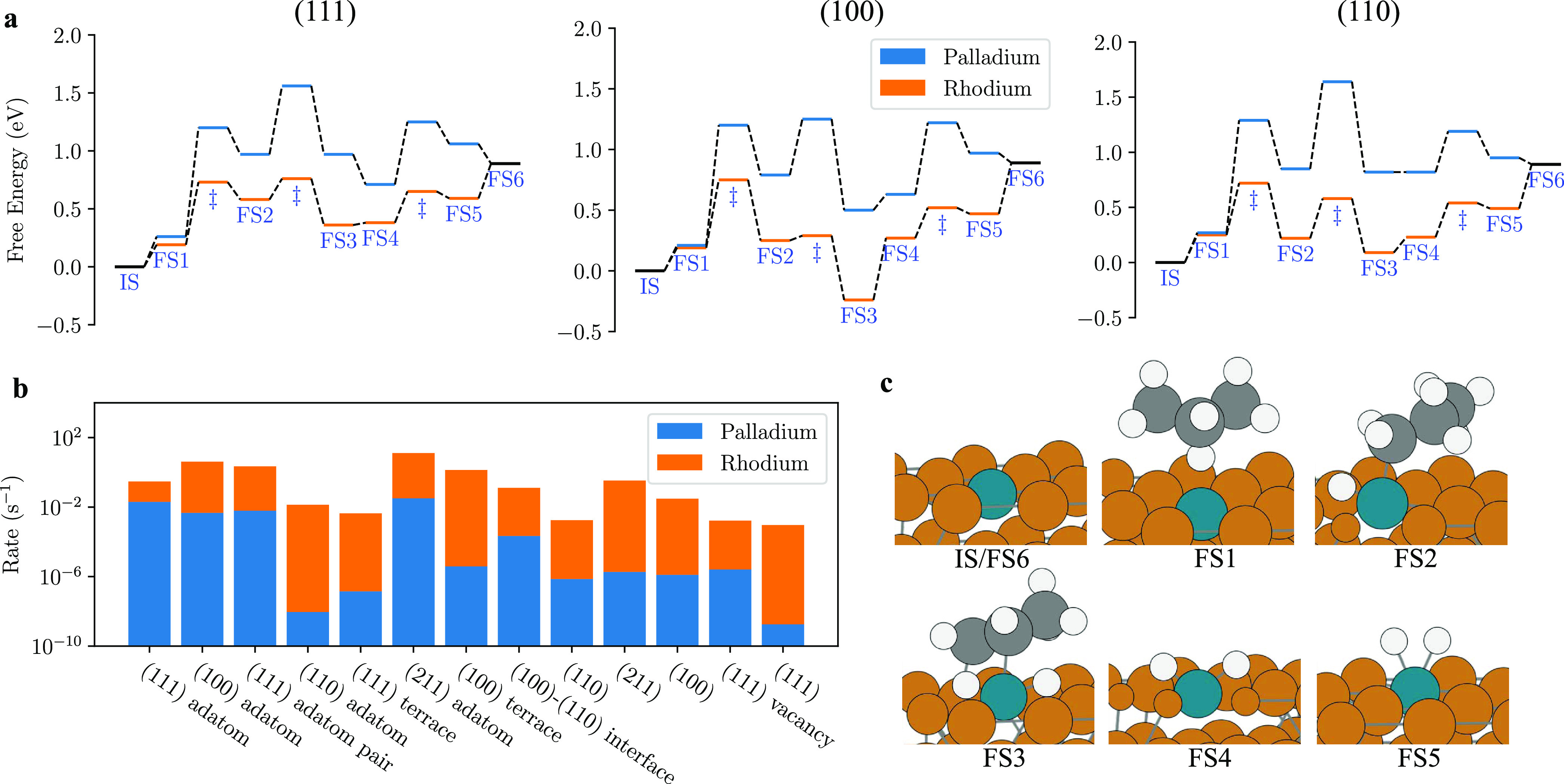
Reactivity and pathways for dehydrogenation of propane. (a) Free energy pathways for the dehydrogenation of propane (for the mechanism that the central carbon atom undergoes C–H activation first) for Rh–Cu and Pd–Cu SAAs for the (111) surface (left), (100) surface (middle), and (110) surface (right). The temperature is set to 400 K, and standard pressure for all gas-phase molecules is used. Initial states (IS) and final states (FS) mark the initial, intermediate, and final states of the process. Transition states are marked with a double dagger symbol. (b) Rate constant for each surface site under 400 K and partial pressures of 0.014 bar of propane and 0.007 bar of hydrogen. (c) Schematic of the mechanism for the central propane atom undergoing C–H activation first for the (111) surface. The pathway that is also considered as part of the microkinetic modeling, the terminal carbon of propane undergoing C–H activation first, is not shown.

The free energy pathway is calculated (Figure 2a) for the catalytic process, giving the energy barriers for each elementary step using a slab model (all sites and energies listed in SI.3). With these energies, microkinetic modeling is subsequently performed. Our methodology enables ML methods and DFT methods to complement each other to effectively describe experimentally representative catalytic systems.

Two pathways are considered for the dehydrogenation of propane: a terminal carbon or the central carbon of propane (where the free energy diagram for the central carbon pathway is shown in Figure 2a and the mechanism in Figure 2c undergoes C–H activation first). First, propane adsorbs to a vacant SAA dopant site on the surface (IS → FS1). The initial C–H activation (FS1 → FS2) is followed by C–H activation of the carbon atom that has not yet undergone C–H activation (FS2 → FS3). Subsequently, the formed propene desorbs from the surface (FS3 → FS4). The surface hydrogen atoms bond together to form molecular hydrogen (FS4 → FS5), which then desorbs from the surface (FS5 → FS6), leaving a vacant surface site. Subsequent surface reactions that affect selectivity are not considered. The reaction is assumed to take place exclusively at the dopant site. Zero-point energy and harmonic free energy corrections are applied for all chemical species.

Rate constants *k* (for each elementary step) are calculated from the free energy barriers for the forward and reverse reactions using harmonic transition state theory, using
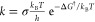
3where σ is the symmetry number factor of the elementary process, Δ*G*^†^ is the free energy change from the initial state to the transition state, *k*_B_ is the Boltzmann constant, *h* is Planck constant, and *T* is the temperature.

During the course of the reaction, the concentration of each surface species/intermediate equilibrates, where a steady state is achieved. Under steady-state conditions, the overall rate of propene formation is calculated. The overall rate is solved at a typical experimental concentration of reactants, at constant pressures of 0.014 and 0.007 bar for propane and hydrogen, respectively. The overall rate of the site is taken at the initial rate of reaction, i.e., the pressure of the product, propene, is considered to be constantly 0 bar.

For both Rh–Cu and Pd–Cu, the rate-determining steps for all sites are the C–H activation steps if it is not propene desorption-limited (Figures 2a and SI.3). Atomic hydrogen coupling to form molecular hydrogen is found to be effectively barrier-less and not significant to the overall pathway in comparison to the C–H activation steps. This is in agreement with other theoretical work.^20,21^ Rh as a dopant atom gives higher reactivity compared to Pd, shown by the higher rate of reaction if a site-per-site comparison is made (Figure 2b). Additionally, the reactivity is loosely related to the number of neighbors the dopant has; the lower the number of neighbors, the lower the C–H activation barrier. Sites with less neighbors are more active. This is most dramatic upon comparing the (111) site (9 neighbors) with the adatom sites. For Rh as the dopant, the (111) adatom site is the order of ∼10^2^ more reactive than the (111) site; for Pd, it is the order of ∼10^7^ more reactive. Comparing the most stable facet, (111), with the second most stable facet, (100): for Rh as a dopant, the (100) site is ∼30× more reactive, while for Pd it is ∼1000× more reactive. The (111) site is the least reactive site for both SAAs.

### Total Reactivity of SAA Nanoparticles

**Figure 3:**
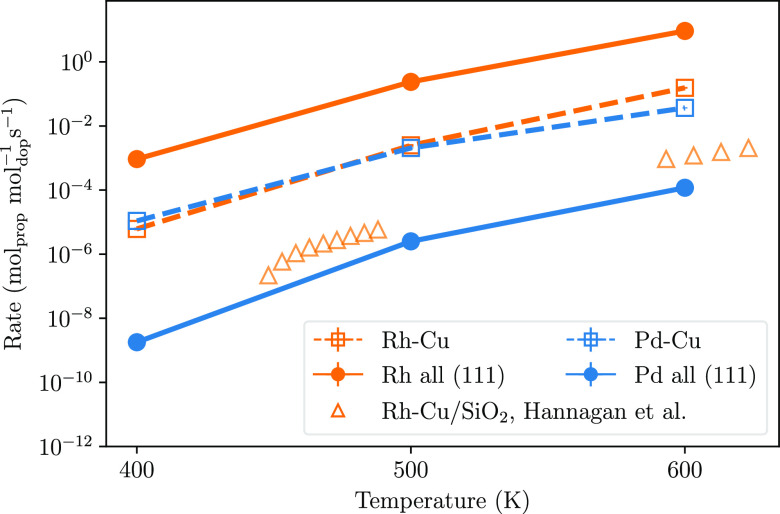
Calculated TOFs for the dehydrogenation of propane to propene using the described Rh–Cu and Pd–Cu SAA nanoparticles. The rate is considered for all single-atom dopants occupying the (111) site (labeled “dopant all (111)”) and for the dopants occupying the sites based on the probability of each site being occupied from calculations in Figure 1b (labeled “dopant–Cu”). The theoretical results are compared to reported experimental data for Rh–Cu adsorbed supported on SiO_2_, reproduced with permission from ref (21). Copyright 2021 The American Association for the Advancement of Science. Statistical error is calculated from the site occupation error as found in Figure 1b.

The total reactivity of the SAA nanoparticles is calculated in two different ways at temperatures of 400, 500, and 600 K, as shown in Figure 3. It is first considered for a traditional microkinetic model where only the most stable (111) surface is considered and all dopant atoms occupy this site. The other scenario considered is where the catalyst is allowed to evolve to its equilibrium structure with respect to temperature, where the ensemble of different sites is considered. This is applied through Equation 2 and the results of Figures 1b and 2b.

For a traditional (111) microkinetic model at 400 K, Rh–Cu has a calculated rate of 9 × 10^–4^ s^–1^ and Pd–Cu has a calculated rate of 2 × 10^–9^ s^–1^ (Figure 3). With a traditional method of modeling, rhodium as a dopant would be indicated to have a drastically higher reactivity than that of palladium. However, when the full dynamics of an SAA nanoparticle is considered as part of the microkinetic model, Rh–Cu is found to have a rate of 6 × 10^–6^ mol_prop_ mol_dop_^–1^ s^–1^ while it is 1 × 10^–5^ mol_prop_ mol_dop_^–1^ s^–1^ for Pd–Cu. While the difference between the calculated rates of the two SAAs is distinct within the calculated statistical error, in view of the error of DFT as a method, the results suggest that the SAAs have similar reactivity. This is due to a traditional microkinetic model that only considers the most stable surface—overestimating the rate for Rh–Cu and underestimating the rate for Pd–Cu.

There are two key reasons for the failure of a model that only considers all of the dopants occupying the (111) surface site. The first reason is that not all of the dopant occupies a site on the surface. At 400 K, only ∼1% of rhodium dopant atoms are at surface sites, while this is ∼86% for palladium as a dopant atom (Figure 1b). This effect is the main reason for the overestimation of the activity of Rh–Cu. The second reason is the prevalence of less stable surface sites that have higher activity. The (111) surface is the least reactive surface for both rhodium and palladium as a dopant (Figure 2b). For rhodium, almost exclusively the (111) sites are occupied, leading to the reactivity of the catalyst being describable by the reactivity of the (111) surface and the number of surface dopants. For palladium, however, the other sites contribute significantly to the overall reactivity of the nanoparticle. The (111) surface only contributes to ∼0.006% of the total reactivity of the Pd–Cu nanoparticle at 400 K. This is counter to Rh–Cu, where (111) sites exclusively contribute to the reactivity of the catalyst.

In comparison to experimental work, the calculated rate for Rh–Cu at 400 K with the full nanoparticle model is within the same order of the recorded experimental result (10^–6^) for the temperature range of 443–488 K.^21^ This overestimation of rate could be caused by underestimation of energy barriers at the DFT level, which is typical of GGA functionals.^58^ It could also be caused by the breakdown of the harmonic approximation at higher temperatures.^59^ The influence of surface species on surface site formation was not considered, yet it does have the potential to have an effect for some single-atom alloys, as seen in other work with the strongly adsorbing molecule, CO.^60^ There is also a needed simplification of the surface site classification by only considering the first nearest neighbors. This causes similar facets, such as the stepped (211) and (311) sites, to be modeled as being equivalent. A marginal effect from the next nearest neighbors for the chemical reactivity of a site would be expected.^61^

For metal surfaces, edge sites and other facets must inevitably exist. It has been demonstrated experimentally that the majority of reaction events will take place at an edge site of a surface.^62^ These nondominant facet sites and edge sites will inevitably exist for pure metal surfaces. However, for SAAs, these sites do not necessarily have to exist. While the bulk metal will have these sites and possibly more reactive defects, they may not be occupied by the dopant. If the occupation of a site is strongly thermodynamically disfavored compared to the most stable site, it will be negligibly occupied. This could give further control over the reactivity and selectivity of a process if the occupation of the dopant is restricted to one kind of site. This could also be observed for changing the size of nanoparticles. The entropy of more bulk sites being available for the dopant to occupy could be overcome by the thermodynamic potential of the dopant occupying a surface site. There may be less dependence on the activity of an SAA catalyst on the size of a nanoparticle. For nanoparticle shape, there may be deactivation of the catalyst dependent on the morphology. For example, Rh–Cu disfavors occupying the (100) site. If a cube-shaped Rh–Cu nanoparticle with only (100) facets is synthesized, less surface dopant sites may form.

## Conclusions

We demonstrate the ability that machine learning techniques have to give a deeper understanding of the assembly of catalytic materials. The combination of DFT calculations with ML potential calculations covers the respective weaknesses of the methods; DFT calculations allow consideration of complex potential energy surfaces for reactions across a variety of different surfaces, while ML potentials allow consideration of the catalyst at the experimental scale. Our results stress the importance that the changing configuration of SAA catalysts and the availability of sites must be considered to better model the reactivity of SAAs.

Beyond modeling the reactivity of catalysts, greater insight into the unique character of SAAs has been given. The special site selectivity that SAAs possess could be exploited in different ways to possibly offer enhanced chemical selectivity if certain surface sites are selective for specific reactants or for forming certain products. This insight could offer new approaches to selecting dopant atoms and host metals for building new single-atom alloy catalysts.

We hope our methods will be used in other catalytic systems of interest, such as formation of catalytic clusters on a support, site locations of supported catalytic single atoms, and surface defect formation.

## Supporting Information Available

Input files, datasets, ML potentials, surface structures, and the packages are available and under development in the repository: https://github.com/FelixWodaczek/surface_classify; with a snapshot also made at DOI: 10.5281/zenodo.8033810 (PDF)
