# Supplementary material for: Reactivity of Single-Atom Alloy Nanoparticles: Modeling the Dehydrogenation of Propane
Source: J Am Chem Soc. 2023 Jun 30;145(27):14894–902. doi: 10.1021/jacs.3c04030 (PMC10347548; doi:10.1021/jacs.3c04030)
Supplement: Supplementary file 1 — ja3c04030_si_001.pdf [file ja3c04030_si_001.pdf]

Supplementary Information – Reactivity of single atom alloy nanoparticles: modelling the dehydrogenation of propane

Rhys J. Bunting,[*,†,‡] Felix Wodaczek,[†,‡] Tina Torabi,[†] and Bingqing Cheng[†]

†*Insitute of Science and Technology, Klosterneuburg, Austria*

‡*These authors contributed equally to this work.*

E-mail: rhys.bunting@ist.ac.at

Contents

1 Surface stability of dopants **4**

1.1 Rh-Cu total energy of bulk to surface dopant diffusion 4

1.2 Pd-Cu total energy of bulk to surface dopant diffusion 4

2 Site classification **5**

2.1 Monte carlo swapping of Rh-Cu and Pd-Cu SAAs 5

2.2 Example structures of sites on the nanoparticle 6

3 Elementary step free energies for Rh-Cu and Pd-Cu surfaces **7**

3.1 Energy barriers for each site of Rh-Cu 8

3.1.1 (111) adatom 8

3.1.2 (100) adatom 9

3.1.3 (111) adatom pair 10

3.1.4 (110) adatom 11

3.1.5 (111) terrace 12

3.1.6 (211) adatom 13

3.1.7 (100) terrace 14

3.1.8 (100)-(110) interface 15

3.1.9 (110) 16

3.1.10 (211) 17

3.1.11 (100) 18

3.1.12 (111) vacancy 19

3.1.13 (111) 20

3.2 Energy barriers for each site of Pd-Cu 21

3.2.1 (111) adatom 21

3.2.2 (100) adatom 22

3.2.3 (111) adatom pair 23
3.2.4 (110) adatom 24
3.2.5 (111) terrace 25
3.2.6 (211) adatom 26
3.2.7 (100) terrace 27
3.2.8 (100)-(110) interface 28
3.2.9 (110) 29
3.2.10 (211) 30
3.2.11 (100) 31
3.2.12 (111) vacancy 32
3.2.13 (111) 33

1 Surface stability of dopants

1.1 Rh-Cu total energy of bulk to surface dopant diffusion

Table S1: Total energy change of diffusion for the (100), (110), and (111) surface for Rh-Cu using a slab model with DFT and a nanoparticle model with the generated ML potential. All energies reported are in electron volts.

Rh-Cu	Δ100	Δ110	Δ111
DFT (Slab)	0.32	0.44	0.05
MLP (Nanoparticle)	0.28	0.44	0.06

1.2 Pd-Cu total energy of bulk to surface dopant diffusion

Table S2: Total energy change of diffusion for the (100), (110), and (111) surface for Rh-Cu using a slab model with DFT and a nanoparticle model with the generated MLP. All energies reported are in electron volts.

Pd-Cu	Δ100	Δ110	Δ111
DFT (Slab)	-0.12	-0.11	-0.21
MLP (Nanoparticle)	-0.17	-0.12	-0.18

2 Site classification

2.1 Monte carlo swapping of Rh-Cu and Pd-Cu SAAs

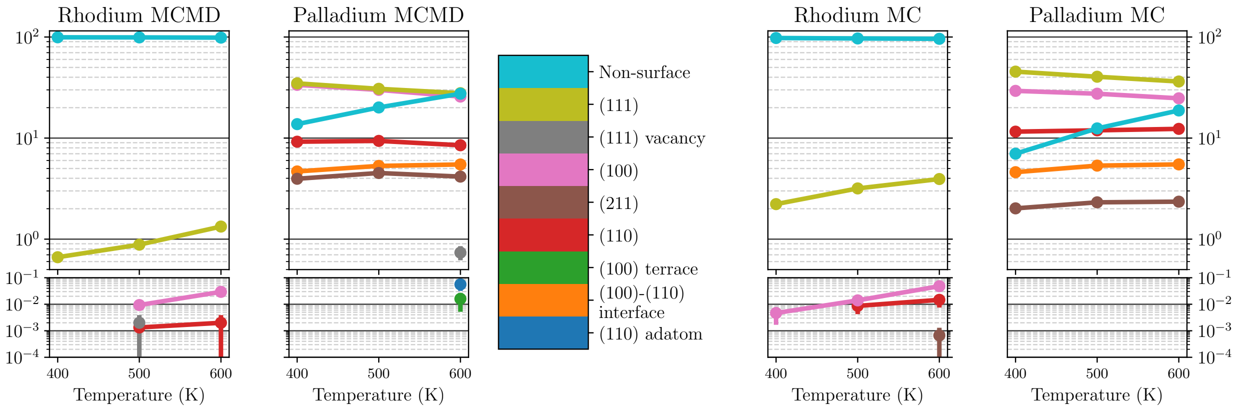

Figure S1: Comparing the dopant occupation of transmutation Monte Carlo swap with molecular dynamics calculations (left) with transmutation Monte Carlo swap calculations (right) of Rh-Cu and Pd-Cu SAAs.

2.2 Example structures of sites on the nanoparticle

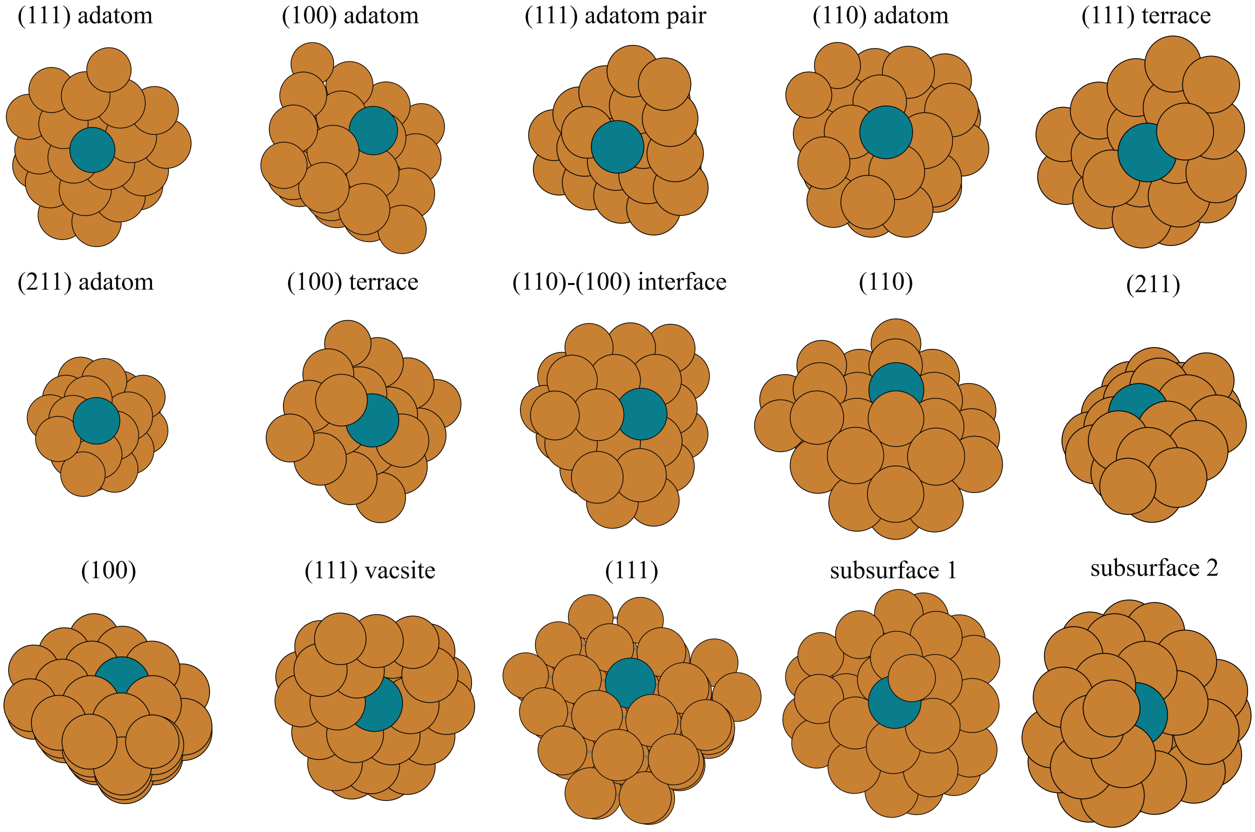

Figure S2: Example local structures of how the first nearest neighbour environments can form as part of the nanoparticle surface.

3 Elementary step free energies for Rh-Cu and Pd-Cu surfaces

The considered elementary steps are labelled as:

1. S1 = $C_3H_{8(g)}$ + * → C_3H_8*
2. S2 = C_3H_8* → C*$H_2CH_2CH_3$ + H*
3. S3 = C_3H_8* → CH_3C*HCH_3 + H*
4. S4 = C*$H_2CH_2CH_3$ + H* → C_3H_6* + 2H*
5. S5 = CH_3C*HCH_3 + H* → C_3H_6* + 2H*
6. S6 = C_3H_6* + 2H* → $C_3H_{6(g)}$ + 2H*
7. S7 = 2H* → H_2*
8. S8 = H_2* → $H_{2(g)}$ + *

All energies are reported in electron volts.

3.1 Energy barriers for each site of Rh-Cu

3.1.1 (111) adatom

0 K	F	B
S1	0.05	0.60
S2	0.19	0.29
S3	0.22	0.58
S4	0.07	0.53
S5	0.16	0.36
S6	1.74	0.05
S7	0.40	0.05
S8	0.83	0.05

400 K	F	B
S1	0.33	0.05
S2	0.06	0.31
S3	0.06	0.58
S4	0.05	0.62
S5	0.07	0.38
S6	0.81	0.05
S7	0.42	0.05
S8	0.36	0.05

500 K	F	B
S1	0.58	0.05
S2	0.06	0.34
S3	0.05	0.59
S4	0.05	0.61
S5	0.08	0.38
S6	0.57	0.05
S7	0.41	0.05
S8	0.25	0.05

600 K	F	B
S1	0.83	0.05
S2	0.06	0.37
S3	0.05	0.61
S4	0.05	0.61
S5	0.08	0.39
S6	0.34	0.05
S7	0.40	0.05
S8	0.14	0.05

3.1.2 (100) adatom

0 K	F	B
S1	0.05	0.59
S2	0.33	0.38
S3	0.36	0.48
S4	0.11	0.48
S5	0.15	0.44
S6	1.71	0.05
S7	0.30	0.05
S8	0.81	0.05

400 K	F	B
S1	0.34	0.05
S2	0.20	0.37
S3	0.20	0.49
S4	0.05	0.52
S5	0.05	0.40
S6	0.80	0.05
S7	0.26	0.05
S8	0.33	0.05

500 K	F	B
S1	0.59	0.05
S2	0.20	0.38
S3	0.18	0.49
S4	0.05	0.52
S5	0.05	0.39
S6	0.57	0.05
S7	0.24	0.05
S8	0.22	0.05

600 K	F	B
S1	0.85	0.05
S2	0.19	0.38
S3	0.16	0.50
S4	0.05	0.53
S5	0.05	0.38
S6	0.33	0.05
S7	0.22	0.05
S8	0.11	0.05

3.1.3 (111) adatom pair

0 K	F	B
S1	0.05	0.82
S2	0.30	0.49
S3	0.33	0.47
S4	0.33	0.42
S5	0.17	0.31
S6	1.85	0.05
S7	0.21	0.05
S8	0.84	0.05

400 K	F	B
S1	0.05	0.07
S2	0.21	0.54
S3	0.26	0.49
S4	0.25	0.45
S5	0.07	0.36
S6	1.00	0.05
S7	0.21	0.05
S8	0.37	0.05

500 K	F	B
S1	0.25	0.05
S2	0.23	0.56
S3	0.28	0.50
S4	0.26	0.46
S5	0.05	0.36
S6	0.77	0.05
S7	0.21	0.05
S8	0.26	0.05

600 K	F	B
S1	0.47	0.05
S2	0.25	0.59
S3	0.30	0.50
S4	0.27	0.47
S5	0.05	0.39
S6	0.56	0.05
S7	0.20	0.05
S8	0.15	0.05

3.1.4 (110) adatom

0 K	F	B
S1	0.05	0.63
S2	0.23	0.49
S3	0.47	0.73
S4	0.05	0.38
S5	0.05	0.37
S6	1.56	0.05
S7	0.90	0.20
S8	0.72	0.05

400 K	F	B
S1	0.28	0.05
S2	0.08	0.49
S3	0.33	0.74
S4	0.05	0.36
S5	0.05	0.36
S6	0.61	0.05
S7	0.78	0.12
S8	0.22	0.05

500 K	F	B
S1	0.51	0.05
S2	0.08	0.51
S3	0.32	0.75
S4	0.05	0.35
S5	0.05	0.34
S6	0.38	0.05
S7	0.77	0.12
S8	0.09	0.05

600 K	F	B
S1	0.75	0.05
S2	0.08	0.53
S3	0.31	0.76
S4	0.05	0.32
S5	0.08	0.35
S6	0.14	0.05
S7	0.77	0.12
S8	0.05	0.14

3.1.5 (111) terrace

0 K	F	B
S1	0.05	0.67
S2	0.44	0.51
S3	0.44	0.63
, S4	0.08	0.42
S5	0.07	0.30
S6	1.49	0.05
S7	0.47	0.05
S8	0.93	0.05

400 K	F	B
S1	0.25	0.05
S2	0.24	0.42
S3	0.27	0.58
S4	0.05	0.45
S5	0.05	0.33
S6	0.55	0.05
S7	0.44	0.05
S8	0.45	0.05

500 K	F	B
S1	0.50	0.05
S2	0.20	0.40
S3	0.24	0.57
S4	0.05	0.45
S5	0.05	0.33
S6	0.31	0.05
S7	0.42	0.05
S8	0.34	0.05

600 K	F	B
S1	0.75	0.05
S2	0.16	0.38
S3	0.22	0.56
S4	0.05	0.46
S5	0.05	0.33
S6	0.07	0.05
S7	0.41	0.05
S8	0.23	0.05

3.1.6 (211) adatom

0 K	F	B
S1	0.05	0.74
S2	0.44	0.32
S3	0.42	0.51
S4	0.07	0.42
S5	0.05	0.18
S6	1.83	0.05
S7	0.07	0.05
S8	0.86	0.05

400 K	F	B
S1	0.19	0.05
S2	0.32	0.37
S3	0.24	0.50
S4	0.05	0.48
S5	0.05	0.26
S6	0.91	0.05
S7	0.10	0.05
S8	0.38	0.05

500 K	F	B
S1	0.43	0.05
S2	0.31	0.38
S3	0.22	0.50
S4	0.05	0.48
S5	0.05	0.26
S6	0.67	0.05
S7	0.09	0.05
S8	0.27	0.05

600 K	F	B
S1	0.69	0.05
S2	0.30	0.40
S3	0.19	0.51
S4	0.05	0.48
S5	0.05	0.26
S6	0.44	0.05
S7	0.08	0.05
S8	0.15	0.05

3.1.7 (100) terrace

0 K	F	B
S1	0.05	0.68
S2	0.50	0.57
S3	0.50	0.38
S4	0.43	0.50
S5	0.18	0.44
S6	1.54	0.05
S7	0.17	0.05
S8	0.92	0.05

400 K	F	B
S1	0.20	0.05
S2	0.36	0.52
S3	0.41	0.41
S4	0.32	0.51
S5	0.09	0.43
S6	0.63	0.05
S7	0.16	0.05
S8	0.44	0.05

500 K	F	B
S1	0.43	0.05
S2	0.35	0.49
S3	0.41	0.42
S4	0.32	0.52
S5	0.10	0.44
S6	0.39	0.05
S7	0.15	0.05
S8	0.33	0.05

600 K	F	B
S1	0.67	0.05
S2	0.33	0.47
S3	0.41	0.43
S4	0.32	0.53
S5	0.11	0.44
S6	0.16	0.05
S7	0.14	0.05
S8	0.21	0.05

3.1.8 (100)-(110) interface

0 K	F	B
S1	0.05	0.72
S2	0.60	0.47
S3	0.61	0.59
S4	0.06	0.46
S5	0.09	0.38
S6	1.66	0.05
S7	0.34	0.13
S8	0.87	0.05

400 K	F	B
S1	0.15	0.05
S2	0.51	0.49
S3	0.52	0.66
S4	0.05	0.52
S5	0.06	0.38
S6	0.80	0.05
S7	0.21	0.05
S8	0.39	0.05

500 K	F	B
S1	0.37	0.05
S2	0.54	0.50
S3	0.53	0.67
S4	0.05	0.54
S5	0.09	0.40
S6	0.58	0.05
S7	0.19	0.05
S8	0.28	0.05

600 K	F	B
S1	0.58	0.05
S2	0.56	0.51
S3	0.55	0.69
S4	0.05	0.55
S5	0.12	0.43
S6	0.37	0.05
S7	0.17	0.05
S8	0.16	0.05

3.1.9 (110)

0 K	F	B
S1	0.05	0.65
S2	0.66	0.48
S3	0.46	0.36
S4	0.37	0.46
S5	0.36	0.38
S6	1.10	0.05
S7	0.34	0.05
S8	0.93	0.05

400 K	F	B
S1	0.30	0.05
S2	0.48	0.51
S3	0.30	0.39
S4	0.37	0.50
S5	0.27	0.35
S6	0.19	0.05
S7	0.31	0.05
S8	0.46	0.05

500 K	F	B
S1	0.55	0.05
S2	0.46	0.52
S3	0.28	0.39
S4	0.39	0.50
S5	0.28	0.35
S6	0.05	0.14
S7	0.29	0.05
S8	0.34	0.05

600 K	F	B
S1	0.81	0.05
S2	0.44	0.54
S3	0.26	0.40
S4	0.41	0.51
S5	0.29	0.35
S6	0.05	0.38
S7	0.28	0.05
S8	0.23	0.05

3.1.10 (211)

0 K	F	B
S1	0.05	0.74
S2	0.43	0.25
S3	0.53	0.37
S4	0.13	0.45
S5	0.07	0.37
S6	1.39	0.05
S7	0.35	0.05
S8	0.94	0.05

400 K	F	B
S1	0.18	0.05
S2	0.25	0.17
S3	0.38	0.35
S4	0.05	0.49
S5	0.05	0.42
S6	0.48	0.05
S7	0.34	0.05
S8	0.44	0.05

500 K	F	B
S1	0.43	0.05
S2	0.21	0.13
S3	0.35	0.35
S4	0.05	0.51
S5	0.05	0.43
S6	0.26	0.05
S7	0.33	0.05
S8	0.33	0.05

600 K	F	B
S1	0.68	0.05
S2	0.17	0.10
S3	0.32	0.35
S4	0.05	0.54
S5	0.05	0.44
S6	0.05	0.06
S7	0.32	0.05
S8	0.21	0.05

3.1.11 (100)

0 K	F	B
S1	0.05	0.67
S2	0.68	0.48
S3	0.53	0.38
S4	0.19	0.53
S5	0.30	0.57
S6	1.46	0.05
S7	0.20	0.05
S8	0.94	0.05

400 K	F	B
S1	0.24	0.05
S2	0.56	0.51
S3	0.38	0.39
S4	0.05	0.54
S5	0.12	0.54
S6	0.56	0.05
S7	0.25	0.05
S8	0.47	0.05

500 K	F	B
S1	0.48	0.05
S2	0.57	0.52
S3	0.36	0.39
S4	0.05	0.55
S5	0.11	0.52
S6	0.34	0.05
S7	0.24	0.05
S8	0.37	0.05

600 K	F	B
S1	0.71	0.05
S2	0.57	0.54
S3	0.33	0.40
S4	0.05	0.57
S5	0.09	0.51
S6	0.12	0.05
S7	0.23	0.05
S8	0.26	0.05

3.1.12 (111) vacancy

0 K	F	B
S1	0.05	0.66
S2	0.85	0.38
S3	0.70	0.27
S4	0.08	0.28
S5	0.17	0.32
S6	1.17	0.05
S7	0.12	0.05
S8	0.90	0.05

400 K	F	B
S1	0.26	0.05
S2	0.71	0.35
S3	0.59	0.24
S4	0.05	0.36
S5	0.05	0.35
S6	0.27	0.05
S7	0.10	0.05
S8	0.41	0.05

500 K	F	B
S1	0.49	0.05
S2	0.71	0.36
S3	0.59	0.24
S4	0.05	0.37
S5	0.05	0.36
S6	0.05	0.05
S7	0.09	0.05
S8	0.30	0.05

600 K	F	B
S1	0.73	0.05
S2	0.71	0.37
S3	0.58	0.25
S4	0.05	0.39
S5	0.05	0.38
S6	0.05	0.26
S7	0.08	0.05
S8	0.18	0.05

3.1.13 (111)

0 K	F	B
S1	0.05	0.67
S2	0.72	0.23
S3	0.78	0.38
S4	0.29	0.43
S5	0.45	0.49
S6	0.98	0.05
S7	0.28	0.05
S8	0.85	0.05

400 K	F	B
S1	0.24	0.05
S2	0.55	0.15
S3	0.66	0.36
S4	0.18	0.40
S5	0.34	0.47
S6	0.07	0.05
S7	0.26	0.05
S8	0.35	0.05

500 K	F	B
S1	0.47	0.05
S2	0.52	0.13
S3	0.66	0.37
S4	0.18	0.40
S5	0.35	0.48
S6	0.05	0.26
S7	0.25	0.05
S8	0.24	0.05

600 K	F	B
S1	0.71	0.05
S2	0.49	0.11
S3	0.66	0.39
S4	0.18	0.40
S5	0.36	0.48
S6	0.05	0.47
S7	0.23	0.05
S8	0.12	0.05

3.2 Energy barriers for each site of Pd-Cu

3.2.1 (111) adatom

0 K	F	B
S1	0.05	0.58
S2	0.89	0.10
S3	0.90	0.15
S4	0.06	0.72
S5	0.08	0.70
S6	1.58	0.05
S7	0.75	0.59
S8	0.47	0.05

400 K	F	B
S1	0.34	0.05
S2	0.69	0.07
S3	0.70	0.19
S4	0.05	0.88
S5	0.05	0.77
S6	0.68	0.05
S7	0.73	0.47
S8	0.05	0.13

500 K	F	B
S1	0.57	0.05
S2	0.66	0.06
S3	0.66	0.19
S4	0.05	0.89
S5	0.07	0.78
S6	0.46	0.05
S7	0.73	0.49
S8	0.05	0.22

600 K	F	B
S1	0.81	0.05
S2	0.61	0.05
S3	0.62	0.21
S4	0.05	0.91
S5	0.08	0.79
S6	0.24	0.05
S7	0.73	0.50
S8	0.05	0.30

3.2.2 (100) adatom

0 K	F	B
S1	0.05	0.56
S2	1.01	0.28
S3	1.08	0.24
S4	0.06	0.37
S5	0.35	0.77
S6	1.48	0.05
S7	0.54	0.52
S8	0.39	0.05

400 K	F	B
S1	0.26	0.05
S2	0.92	0.32
S3	0.98	0.27
S4	0.05	0.47
S5	0.23	0.75
S6	0.57	0.05
S7	0.50	0.48
S8	0.05	0.09

500 K	F	B
S1	0.44	0.05
S2	0.94	0.34
S3	0.99	0.29
S4	0.05	0.46
S5	0.24	0.76
S6	0.34	0.05
S7	0.49	0.49
S8	0.05	0.15

600 K	F	B
S1	0.61	0.05
S2	0.95	0.36
S3	1.00	0.32
S4	0.05	0.47
S5	0.25	0.76
S6	0.11	0.05
S7	0.49	0.51
S8	0.05	0.20

3.2.3 (111) adatom pair

0 K	F	B
S1	0.05	0.63
S2	0.92	0.16
S3	1.02	0.38
S4	0.37	0.73
S5	0.51	0.74
S6	1.71	0.05
S7	0.34	0.52
S8	0.46	0.05

400 K	F	B
S1	0.21	0.05
S2	0.79	0.22
S3	0.88	0.40
S4	0.32	0.76
S5	0.40	0.75
S6	0.84	0.05
S7	0.24	0.45
S8	0.06	0.05

500 K	F	B
S1	0.41	0.05
S2	0.79	0.24
S3	0.87	0.41
S4	0.35	0.77
S5	0.39	0.74
S6	0.62	0.05
S7	0.22	0.46
S8	0.05	0.10

600 K	F	B
S1	0.61	0.05
S2	0.78	0.27
S3	0.87	0.41
S4	0.38	0.78
S5	0.38	0.73
S6	0.41	0.05
S7	0.20	0.47
S8	0.05	0.17

3.2.4 (110) adatom

0 K	F	B
S1	0.05	0.57
S2	1.07	0.30
S3	1.16	0.45
S4	0.06	0.66
S5	0.11	0.66
S6	1.08	0.05
S7	1.16	0.50
S8	0.41	0.05

400 K	F	B
S1	0.28	0.05
S2	0.87	0.23
S3	1.02	0.46
S4	0.05	0.72
S5	0.09	0.68
S6	0.18	0.05
S7	1.06	0.48
S8	0.05	0.06

500 K	F	B
S1	0.48	0.05
S2	0.85	0.20
S3	1.02	0.48
S4	0.05	0.75
S5	0.12	0.71
S6	0.05	0.15
S7	1.06	0.51
S8	0.05	0.12

600 K	F	B
S1	0.68	0.05
S2	0.83	0.18
S3	1.01	0.50
S4	0.05	0.77
S5	0.15	0.74
S6	0.05	0.37
S7	1.06	0.53
S8	0.05	0.18

3.2.5 (111) terrace

0 K	F	B
S1	0.05	0.63
S2	1.13	0.14
S3	1.19	0.22
S4	0.35	0.62
S5	0.23	0.48
S6	0.81	0.05
S7	0.47	0.22
S8	0.60	0.05

400 K	F	B
S1	0.22	0.05
S2	1.04	0.24
S3	1.08	0.34
S4	0.27	0.62
S5	0.15	0.44
S6	0.05	0.13
S7	0.39	0.15
S8	0.15	0.05

500 K	F	B
S1	0.42	0.05
S2	1.07	0.28
S3	1.10	0.38
S4	0.27	0.62
S5	0.14	0.42
S6	0.05	0.34
S7	0.38	0.16
S8	0.07	0.05

600 K	F	B
S1	0.62	0.05
S2	1.09	0.32
S3	1.12	0.43
S4	0.26	0.62
S5	0.12	0.40
S6	0.05	0.56
S7	0.38	0.16
S8	0.05	0.11

3.2.6 (211) adatom

0 K	F	B
S1	0.05	0.69
S2	1.07	0.17
S3	1.07	0.23
S4	0.41	0.73
S5	0.18	0.45
S6	1.42	0.05
S7	0.43	0.46
S8	0.49	0.05

400 K	F	B
S1	0.14	0.05
S2	0.91	0.17
S3	0.91	0.20
S4	0.31	0.72
S5	0.10	0.47
S6	0.45	0.05
S7	0.44	0.45
S8	0.12	0.05

500 K	F	B
S1	0.34	0.05
S2	0.89	0.15
S3	0.89	0.19
S4	0.31	0.70
S5	0.11	0.48
S6	0.20	0.05
S7	0.45	0.48
S8	0.07	0.05

600 K	F	B
S1	0.54	0.05
S2	0.86	0.14
S3	0.87	0.18
S4	0.30	0.69
S5	0.13	0.49
S6	0.05	0.15
S7	0.46	0.52
S8	0.05	0.08

3.2.7 (100) terrace

0 K	F	B
S1	0.05	0.69
S2	1.08	0.34
S3	1.37	0.28
S4	0.97	0.92
S5	0.39	0.69
S6	1.16	0.05
S7	0.34	0.26
S8	0.42	0.05

400 K	F	B
S1	0.13	0.05
S2	0.97	0.38
S3	1.24	0.30
S4	0.90	0.95
S5	0.30	0.70
S6	0.28	0.05
S7	0.28	0.20
S8	0.05	0.09

500 K	F	B
S1	0.31	0.05
S2	0.97	0.38
S3	1.24	0.31
S4	0.91	0.96
S5	0.31	0.71
S6	0.05	0.05
S7	0.27	0.21
S8	0.05	0.14

600 K	F	B
S1	0.49	0.05
S2	0.98	0.39
S3	1.24	0.31
S4	0.92	0.97
S5	0.32	0.72
S6	0.05	0.27
S7	0.26	0.22
S8	0.05	0.20

3.2.8 (100)-(110) interface

0 K	F	B
S1	0.05	0.69
S2	1.10	0.27
S3	1.22	0.37
S4	0.49	0.79
S5	0.38	0.71
S6	1.24	0.05
S7	0.57	0.37
S8	0.48	0.05
400 K	F	B
S1	0.13	0.05
S2	0.92	0.19
S3	1.10	0.37
S4	0.35	0.77
S5	0.31	0.72
S6	0.37	0.05
S7	0.47	0.33
S8	0.10	0.05
500 K	F	B
S1	0.31	0.05
S2	0.90	0.16
S3	1.10	0.37
S4	0.34	0.77
S5	0.33	0.75
S6	0.14	0.05
S7	0.46	0.35
S8	0.05	0.05
600 K	F	B
S1	0.49	0.05
S2	0.87	0.12
S3	1.09	0.38
S4	0.32	0.78
S5	0.36	0.77
S6	0.05	0.19
S7	0.45	0.37
S8	0.05	0.09

3.2.9 (110)

0 K	F	B
S1	0.05	0.57
S2	1.24	0.43
S3	1.09	0.34
S4	0.85	0.80
S5	0.77	0.66
S6	0.93	0.05
S7	0.48	0.34
S8	0.41	0.05

400 K	F	B
S1	0.32	0.05
S2	1.03	0.44
S3	0.91	0.37
S4	0.78	0.81
S5	0.63	0.61
S6	0.05	0.05
S7	0.37	0.24
S8	0.05	0.11

500 K	F	B
S1	0.52	0.05
S2	1.00	0.45
S3	0.88	0.37
S4	0.79	0.82
S5	0.62	0.60
S6	0.05	0.29
S7	0.35	0.24
S8	0.05	0.17

600 K	F	B
S1	0.73	0.05
S2	0.98	0.46
S3	0.86	0.38
S4	0.81	0.83
S5	0.61	0.59
S6	0.05	0.52
S7	0.34	0.24
S8	0.05	0.22

3.2.10 (211)

0 K	F	B
S1	0.05	0.69
S2	0.95	0.14
S3	1.21	0.43
S4	0.60	0.80
S5	0.48	0.66
S6	1.05	0.05
S7	0.59	0.29
S8	0.49	0.05

400 K	F	B
S1	0.16	0.05
S2	0.81	0.14
S3	1.05	0.41
S4	0.44	0.78
S5	0.32	0.62
S6	0.19	0.05
S7	0.50	0.22
S8	0.08	0.05

500 K	F	B
S1	0.36	0.05
S2	0.80	0.14
S3	1.03	0.41
S4	0.43	0.77
S5	0.30	0.61
S6	0.05	0.13
S7	0.48	0.23
S8	0.05	0.08

600 K	F	B
S1	0.56	0.05
S2	0.78	0.15
S3	1.00	0.40
S4	0.41	0.76
S5	0.29	0.61
S6	0.05	0.35
S7	0.47	0.25
S8	0.05	0.13

3.2.11 (100)

0 K	F	B
S1	0.05	0.58
S2	1.15	0.39
S3	1.11	0.38
S4	0.62	0.73
S5	0.72	0.80
S6	1.03	0.05
S7	0.60	0.30
S8	0.36	0.05

400 K	F	B
S1	0.26	0.05
S2	0.98	0.41
S3	0.93	0.40
S4	0.46	0.74
S5	0.58	0.83
S6	0.17	0.05
S7	0.60	0.25
S8	0.05	0.13

500 K	F	B
S1	0.45	0.05
S2	0.97	0.41
S3	0.91	0.40
S4	0.44	0.75
S5	0.60	0.85
S6	0.05	0.14
S7	0.59	0.27
S8	0.05	0.18

600 K	F	B
S1	0.63	0.05
S2	0.97	0.41
S3	0.89	0.41
S4	0.43	0.76
S5	0.62	0.88
S6	0.05	0.35
S7	0.59	0.28
S8	0.05	0.23

3.2.12 (111) vacancy

0 K	F	B
S1	0.05	0.57
S2	1.20	0.27
S3	1.13	0.23
S4	0.65	0.63
S5	0.67	0.61
S6	0.85	0.05
S7	0.30	0.16
S8	0.39	0.05

400 K	F	B
S1	0.27	0.05
S2	1.01	0.22
S3	1.02	0.23
S4	0.50	0.63
S5	0.39	0.51
S6	0.05	0.09
S7	0.23	0.10
S8	0.05	0.13

500 K	F	B
S1	0.46	0.05
S2	0.98	0.20
S3	1.02	0.24
S4	0.51	0.64
S5	0.37	0.50
S6	0.05	0.31
S7	0.22	0.12
S8	0.05	0.19

600 K	F	B
S1	0.64	0.05
S2	0.95	0.19
S3	1.02	0.24
S4	0.52	0.65
S5	0.35	0.49
S6	0.05	0.53
S7	0.22	0.13
S8	0.05	0.25

3.2.13 (111)

0 K	F	B
S1	0.05	0.58
S2	1.18	0.31
S3	1.22	0.41
S4	0.72	0.62
S5	0.85	0.70
S6	0.68	0.05
S7	0.67	0.28
S8	0.30	0.05

400 K	F	B
S1	0.31	0.05
S2	0.94	0.23
S3	1.01	0.37
S4	0.60	0.60
S5	0.73	0.67
S6	0.05	0.31
S7	0.54	0.19
S8	0.05	0.22

500 K	F	B
S1	0.52	0.05
S2	0.89	0.21
S3	0.99	0.38
S4	0.61	0.61
S5	0.75	0.67
S6	0.05	0.53
S7	0.52	0.19
S8	0.05	0.28

600 K	F	B
S1	0.72	0.05
S2	0.84	0.19
S3	0.95	0.39
S4	0.62	0.61
S5	0.78	0.68
S6	0.05	0.76
S7	0.50	0.19
S8	0.05	0.34
